# Stroke prevention during and after transcatheter aortic valve implantation: From cerebral protection devices to antithrombotic management

**DOI:** 10.3389/fcvm.2022.958732

**Published:** 2022-10-17

**Authors:** Victor Alfonso Jimenez Diaz, Rodrigo Estevez Loureiro, Jose Antonio Baz Alonso, Pablo Juan Salvadores, Guillermo Bastos Fernandez, Berenice Caneiro Queija, Cesar Veiga Garcia, Andres Iñiguez Romo

**Affiliations:** ^1^Department of Cardiology, Hospital Álvaro Cunqueiro, University Hospital of Vigo, Vigo, Spain; ^2^Cardiovascular Research Group, Galicia Sur Health Research Institute (IIS Galicia Sur), Vigo, Spain

**Keywords:** transcatheter aortic valve implantation (TAVI), stroke, cerebral embolic protection devices, complication, antithrombotic therapy, aortic stenosis (AS)

## Abstract

Since its conception, transcatheter aortic valve implantation (TAVI) has undergone important improvements both in the implantation technique and in transcatheter devices, allowing an enthusiastic adoption of this therapeutic approach in a wide population of patients previously without a surgical option and managed conservatively. Nowadays, patients with severe symptomatic aortic stenosis are typically managed with TAVI, regardless of their risk to surgery, improving the prognosis of patients and thus achieving an exponential global expansion of its use. However, thromboembolic and hemorrhagic complications remain a latent concern in TAVI recipients. Both complications can appear simultaneously in the periprocedural period or during the follow-up, and when minor, they resolved without apparent sequelae, but in a relevant percentage of cases, they are devastating, overshadowing the benefit achieved with TAVI. Our review outlines the etiology and incidence of thromboembolic complications associated with TAVI, the main current strategies for their prevention, and the implications of its pharmacological management at the follow-up in a TAVI population, mostly frail and predisposed to bleeding complications.

## Introduction

The current American (1) and European (2) guidelines for the treatment of patients with valvular heart disease favor transcatheter aortic valve implantation (TAVI) by transfemoral access for patients with aortic stenosis (AS) who are at low to high risk for surgical aortic valve replacement (SAVR). Although the results of its pivotal clinical trials and reports of its clinical use were published, several concerns regarding its neurological safety arose soon afterward.

The expansion of TAVI indication to a younger and less comorbid population has prompted active research into the mechanisms involved in procedure-related stroke and the development of various devices to protect the brain from the passage of emboli during TAVI. Also, the determination of the most balanced antithrombotic strategy after TAVI in terms of ischemic protection and bleeding is a relevant clinical need and is under current quest. In this article, we provide an updated overview on stroke related to TAVI and its most relevant advances in devices aimed at stroke prevention, and on the ongoing clinical research in preventive pharmacological strategies.

## Etiology, timing, and mechanism of transcatheter aortic valve implantation-related stroke

Despite great advances in patient management and latest iterations on TAVI devices occurred during the last decade, stroke has remained steady over time and continues to be a frequent TAVI complication with relevant prognostic implications (Table 1). In general, strokes related to TAVI can be divided into procedure-related strokes (acute) and patient- or prosthesis-related strokes (long-term). In addition, the clinical manifestations can be broad, ranging from silent or subclinical events detected as findings in brain imaging studies, to episodes of transitory delirium or acute confusional state, to a major stroke with manifest clinical expression and disabling sequelae. Overt stroke is one of the most fearful and catastrophic complication of TAVI, being strongly associated with morbidity and mortality (3), increasing the average 30-day mortality more than six times in patients who suffer from it than those who do not after TAVI (4). Also, bleeding complications remain a problem to be solved, not only those that occur periprocedural but also those that continue for a long-standing period, the former being more in relation to TAVI vascular access, and the latter to long-term post-TAVI antithrombotic management (5).

**TABLE 1:** Rates of stroke/TIA in the pivotal randomized TAVI trials.

				Stroke or TIA (%)	
Trial	Year	Sample size	STS-PROM score (mean ± SD)	30 days	1 year
PARTNER 1A	2007–2009	348	11.8 ± 3.3	5.5	8.3
PARTNER 1B	2007–2009	179	11.2 ± 5.8	6.7	10.6
U.S. CoreValve	2011–2012	390	7.3 ± 3.0	5.7	10.4
PARTNER 2A	2011–2013	1,011	5.8 ± 2.1	6.4	10.4
SURTAVI	2012–2016	864	4.4 ± 1.5	4.5	8.2
PARTNER 3	2016–2017	496	1.9 ± 0.7	0.6	2.2
Evolut Low Risk	2016–2018	734	1.9 ± 0.7	4.0	5.8

TAVI, transcatheter aortic valve implantation; STS-PROM, society of thoracic surgeons predicted risk of mortality; TIA, transient ischemic attack.

### Clinical stroke

**FIGURE 1:**
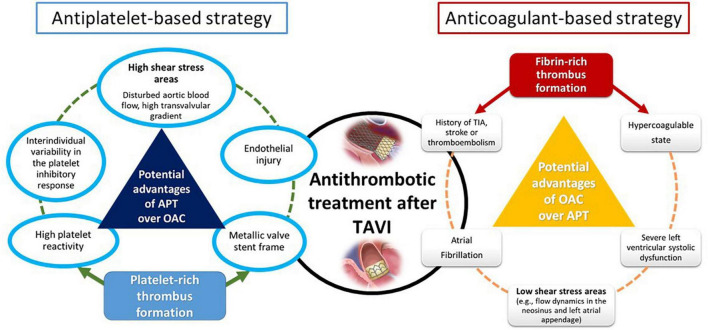
Potential mechanisms related to stroke after TAVI and main antithrombotic strategies. TAVI, transcatheter aortic valve implantation; APT, antiplatelet therapy; OAC, oral anticoagulation therapy; TIA, transient ischemic attack.

Stroke occurrence during and after TAVI is likely multifactorial and closely linked with the patients’ risk profile (6). Ischemic stroke can happen during or after TAVI, either in periprocedural days or during the long-term follow-up; is strongly linked to morbidity; and can entirely nullify TAVI prognostic improvements (5–8). TAVI and transcatheter valve components induce a prothrombotic environment in the aortic root (9, 10). Mechanical disruption of atheromatous or calcific debris during different procedural steps of TAVI (crossing of catheters and devices in the aortic arch/valve, during balloon aortic valvuloplasty, during deployment, or during valve post-dilation) may account as the main mechanisms for most of the periprocedural strokes (11, 12). Also, suboptimal intraprocedural anticoagulation levels inducing the formation of thrombi in guidewires and catheters, air embolism, and severe hypotension states may also be involved in the stroke pathophysiology during TAVI. The use of cerebral embolic protection devices (CEPDs) during TAVI may contribute to decrease the procedural stroke risk. Different biological responses to the presence of an aortic bioprosthesis and its materials, such as increased platelet activation and an acute rise in prothrombotic factors, increased shear stress and endothelial injury, altered aortic flow dynamics in the neosinus, and suboptimal antiplatelet effect, may favor the formation of thrombi and embolic phenomena during the first year after TAVI, with the first 3 months being the period of greatest risk (9, 10, 12–14). Also, postprocedural subacute and late events can be at least partly explained by atrial fibrillation (AF), which has been reported in about 20–40% of patients admitted for TAVI, and by the development of new-onset atrial fibrillation (NOAF) in up to 8% of cases during or after the intervention (15–17). In addition to AF, it is likely that the mechanism of late events is also associated with other baseline characteristics known predictors of late cardiovascular events, such as cerebrovascular disease, peripheral artery disease, and/or renal disease, namely the baseline burden of the aged TAVI patient. Therefore, TAVI-related stroke seems to be linked to both increased platelet activation due to endothelial injury after valve deployment and to AF-related thromboembolic risk factors (Figure 1).

### Subclinical stroke

Different studies have shown the presence of silent cerebral embolic lesions in most patients undergoing percutaneous and surgical aortic valve replacement detected by diffusion-weighted magnetic resonance imaging (DW-MRI) (18, 19). Increasing evidence indicates that these subclinical phenomena may be associated with progressive cognitive deterioration, leading to a neurocognitive decline and dementia (20, 21). The long-term relevance of these clinically “silent” brain lesions still remains unknown, but since they can be found in the vast majority of patients undergoing TAVI, they constitute the hidden part of the iceberg; so, the adoption of preventive measures will be of utmost importance. The recognition of overt strokes and asymptomatic brain lesions after TAVI is largely dependent on the intensity of the neurological and imaging tests used. Therefore, the inclusion of an experienced neurologist in the heart team to assess the neurological integrity after TAVI and to detect any early subtle sign of brain damage is paramount.

## Preventive strategies for acute and late strokes in transcatheter aortic valve implantation

Since manipulation of the transcatheter valve in the calcified aortic arch and native aortic valve plays an important role in the genesis of emboli and periprocedural acute stroke (procedure associated origin) (11, 12), cerebral protection devices may provide benefit (22–25). By contrast, strategies for the prevention of subsequent stroke are based on an optimal and balanced antithrombotic therapy to prevent ischemic events without substantially increasing the risk of long-term bleeding.

### Cerebral embolic protection devices

Given that most CVEs in patients undergoing TAVI are embolic in nature, the use of CEPDs seems reasonable to reduce debris or embolic material that travels to the brain, subsequently minimizing the risk of stroke and lessen the extent of neurological damage. Previous studies have shown the feasibility and safety of CEPD use (22–25), but its efficacy remains to be clearly demonstrated (26).

**FIGURE 2:**
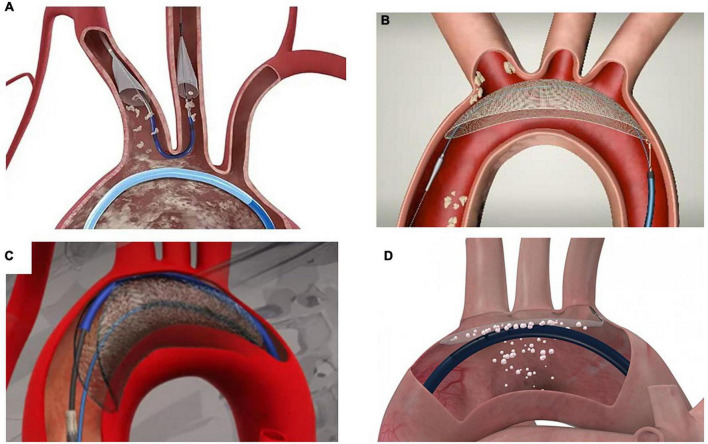
Cerebral embolic protection devices with published data. **(A)** Sentinel^®^ Cerebral Protection System (Boston Scientific, Marlborough, Massachusetts). **(B)** TriGUARD 3 Cerebral Protection Device (Keystone Heart, Tampa, FL, USA, a Venus Medtech Company). **(C)** Emblok Embolic Protection System (Innovative Cardiovascular Solutions, Grand Rapids, Michigan). **(D)** ProtEmbo^®^ Cerebral Protection System (Protembis GmbH, Aachen, Germany).

Currently, there are four devices with published data, two of them under clinical use, and the other two under active investigation in early phase studies for their potential applications in TAVI and structural heart interventions (Figure 2). Basically, they are divided into two types based on their mechanism of action: devices that capture (totally or partially) debris before it reaches the brain arteries and devices that deflect the debris away from the main arterial branches of the aortic arch.

Another classification of devices is based on the brain protection they offer, being partial – covering two of the three main arteries of the aortic arch (brachiocephalic trunk or right common carotid artery and left common carotid artery) – or total (the former two plus the left vertebral artery originating from the left subclavian artery which merges with the right vertebral artery to form the basilar artery, the major supply to the posterior portion of the circle of Willis). The implications of leaving the left subclavian artery and thus the left vertebral artery unprotected are relevant.

Fanning JP and col (27) provided a detailed description of the anatomical distribution and the subsequent cerebral predilection for injury of the cerebral ischemic lesions occurring secondary to TAVI using DW-MRI. The authors observed that the distribution of lesions suggests the posterior circulation and the right hemisphere are particularly vulnerable to perioperative cerebrovascular injury. They found that 59% of all cerebral infarcts occurred in the posterior circulation, and around two-thirds of all lesions affected the right hemisphere. Interestingly, when considering the total volume of infarction, 10,255 μl (90%) occurred in the posterior compared with 1,192 μl (10%) in the anterior circulation (27).

Thus, embolic protection devices that lack coverage of the left subclavian artery also fail to completely protect the posterior circulation, resulting in potentially 19 of 28 cerebral vascular territories and 26% of the brain volume being completely unprotected. The authors hypothesized that the relatively impaired cerebral autoregulation in the posterior versus anterior circulation is a plausible explanation for the observed differences in vulnerability to injury, increasing the importance of providing complete cerebral protection in cases of cardiovascular procedures with risk of cerebral embolization (27).

### Sentinel cerebral protection system

The Sentinel^®^ Cerebral Protection System (Boston Scientific, Marlborough, MA, USA) consists of two polyurethane filters with 140-mm-diameter pores fixed in a flexible nitinol radiopaque frame, advanced from a 6-Fr sheath through the right radial or right brachial artery and deployed into the ostia of the brachiocephalic trunk and left common carotid artery (22). It is designed to capture emboli passing to the cerebral circulation in two of the three branches of the aortic arch, leaving the left subclavian open and potentially the left vertebral circulation unprotected (22, 28). The device has CE and FDA approval and is to date the most widely used CEPD in TAVI.

The MISTRAL-C trial (27) (*n* = 65 patients) and the CLEAN TAVI trial (22) (*n* = 100 patients) are randomized clinical trials that showed fewer new lesions and a smaller total lesion volumes in the protected group with Sentinel vs. no protection. Also, neurocognitive deterioration was more frequent in patients treated without protection (28).

The SENTINEL U.S. IDE trial (25) was a multicenter study (*n* = 363 patients) with a 1:1:1 randomization into a safety device arm (*n* = 123), an imaging device arm (*n* = 121), and an imaging control arm (*n* = 119). The authors found debris in 99% of the filters. Despite a reduction in all-cause strokes at 30 days, statistical significance was not met (5.6% for the EPD group vs. 9.1% in the control group; *p* = 0.25). Also, the decrease in the median total new lesion volume in protected territories (44%) evaluated by DW-MRI 2–7 days after TAVI was not statistically significant (102.8 mm^3^, IQR 36.9–423.2 mm^3^ in the device arm vs. 178.0 mm^3^, IQR 34.3–482.5 mm^3^ in the control arm; *p* = 0.33). It is noteworthy that using the procedural stroke classification by NeuroARC definition, the CEPD group showed a significant reduction in stroke within 72 h after TAVI when compared with the unprotected group (3.0 vs. 8.2%; *p* = 0.053).

In total, two large ongoing randomized trials will probably bring definitive evidence on the efficacy of Sentinel on stroke prevention in TAVI: Stroke PROTECTion With SEntinel During Transcatheter Aortic Valve Replacement (PROTECTED TAVR) (NCT04149535, *N* = 3,000) and British Heart Foundation Randomised Trial of Routine Cerebral Embolic Protection in Transcatheter Aortic Valve Implantation (BHF PROTECT-TAVI) (ISRCTN16665769, *N* = 7,730).

### TriGUARD 3™ cerebral protection device

The TriGUARD 3™ Cerebral Protection Device (Keystone Heart, Tampa, FL, USA, a Venus Medtech Company) is a deflection device positioned in the aortic arch to provide protection to all three branches of the aortic arch, including the left subclavian artery (23). It is placed through a transfemoral access via a 9-Fr femoral arterial sheath, which also allows for concomitant use of a 6-Fr pigtail catheter. The device is composed of a semi-permeable nitinol mesh with pores of 115 × 145 mm, which deflects particles larger than 140 μm (24).

The first and latest generation of the TriGUARD was assessed in four prospective clinical studies of TAVI recipients in the United States and Europe showing a numerical reduction (non-statistically significant) in stroke rates and a lower total lesion volume in cases who have complete coverage of all brain branches than in cases who were not protected in a combined analysis (23, 24).

The feasibility and safety of this device were investigated in DEFLECT I (29) and DEFLECT II (30) trials, which were prospective, single-arm studies (*n* = 37 patients and 14 patients, respectively). Data on DW-MRI showed that in the DEFLECT I trial (28), the presence of new brain infarcts was comparable with those in historical controls (82 vs. 76%, *p* = NS). However, as compared with historical data, the total lesion volume per patient was 34% smaller (0.2 vs. 0.3 cm^3^). Similarly, the DEFLECT II study (30) comparing the DWI-MRI of these patients with that of a historical control group revealed no significant reduction in the number of lesions [median 5.5 vs. 5.0, *p* = 0.857] but a substantial reduction in the mean lesion volume per patient [median 13.8 vs. 25.1, *p* = 0.049].

The DEFLECT III trial (31) (*n* = 85 patients) was a single-blind multicenter randomized trial in which patients with TAVI were randomized to either EPD (*n* = 46) with TriGUARD HDH or no CEPD (*n* = 39). DW-MRI was performed in all patients on days 4 ± 2 and 30 ± 7 after TAVI, as well as multiple serial neurological assessments. The dropout rate for DW-MRI assessment at 4 days was 30% (33 of 46 in the CEPD group and 26 of 39 in the no-CEPD group). Device success was achieved in 88.9% of the patients (40 of 45). The primary in-hospital procedural safety endpoints (death, stroke, life-threatening or disabling bleeding, stage 2 or 3 acute renal failure, or major vascular complications) did not differ statistically in TriGUARD HDH (21.7%) compared with the control group (30.8%, *p* = 0.34), but in cases with complete brain protection, TriGUARD HDH was associated with a higher rate of freedom from new cerebral lesions at 1 month (26.9 vs. 11.5%, p not reported) and less neurological damage assessed by the National Institutes of Health Stroke Scale (NIHSS) (3.1 vs. 15.4%, *p* = 0.16).

The REFLECT I trial (*n* = 258 patients of the initially planned 375 patients) was a multicenter (20 U.S. and 6 European centers), randomized controlled trial that evaluated the safety, efficacy, and performance of the TriGUARD™ HDH device in patients undergoing TAVI (23). There were 54 roll-in patients and 204 patients randomized 2:1 to TriGUARD HDH device (*n* = 141) or control (*n* = 63). The trial was suspended by recommendation of the data safety monitoring committee before patients’ enrollment was completed. The primary efficacy endpoint was a hierarchical composite of (i) all-cause mortality or any stroke at 30 days, (ii) NIHSS worsening at 2–5 days or Montreal Cognitive Assessment worsening at 30 days, and (iii) total volume of brain ischemic lesions detected by DW-MRI at 2–5 days. Complete protection of all three cerebral vessels throughout the TAVI procedure was achieved in 57.3% (78/136).

Compared with the performance goal, the primary safety outcome was met (21.8 vs. 34.4%, *p* < 0.0001). The primary hierarchical efficacy endpoint was comparable between the groups, with a mean score (higher is better) of −5.3 ± 99.8 for TriGUARD and 11.8 ± 96.4 for controls (*p* = 0.314), corresponding to a win percentage of 44.6% for TriGUARD and 55.4% without protection. Comparable results were obtained in patients with complete cerebral coverage (mean score of −2.0 ± 71.4 for TriGUARD and 2.5 ± 70.1 for controls, *p* = 0.766, with a similar win percentage of 48 vs. 52%). When compared with the controls, covert central nervous system damage was reduced with TriGUARD both in-hospital (46.1 vs. 60.3%, *p* = 0.0698) and at 5 days (61.7 vs. 76.2%, *p* = 0.054).

In 18 U.S. sites, the REFLECT II U.S. trial (24) enrolled 220 of the 345 patients planned (63.8%), with 41 roll-in and 179 randomized patients (121 TriGUARD 3 and 58 control subjects). The study suffered an early discontinuation of the enrollment by the sponsor after the U.S. Food and Drug Administration advised for unblinded safety data assessment. Complete cerebral coverage (before, during, and after TAVI) was achieved in 59.7% (94/157), and device interaction was reported in 9.6% (15/157). The primary safety endpoint was met compared with the performance goal (15.9 vs. 34.4%; *p* < 0.0001), but the primary hierarchal efficacy endpoint at 30 days (death or stroke at 30 days, NIHSS score worsening in-hospital, and cerebral ischemic lesions on DW-MRI at 2 to 5 days) was not met (mean scores [higher is better]: −8.58 TG3 vs. 8.08 control; *p* = 0.857).

### Emblok^®^ embolic protection system

The Emblok Embolic Protection System (Innovative Cardiovascular Solutions, Grand Rapids, MI, USA) is a device designed to protect all supraaortic vessels by a full circumferential coverage of the aortic arch (32). The delivery system is an 11-Fr catheter compatible to be deployed through a single access site supported by 0.035 guidewire and integrates a 4-Fr radiopaque pigtail catheter for aortogram performance. Anatomical criteria for its use include an ascending aorta length ≥ 9 cm, an ascending aorta or aortic arch diameter between 30 and 35 mm, and an arterial femoral access suitable for an 11-Fr delivery system. The filter is made of a polyurethane mesh with a pore size of 125 μm, supported by a nitinol frame positioned just proximal to the brachiocephalic trunk. Once the transcatheter valve is deployed, the Emblok system must be recaptured to be able to retrieve the transcatheter delivery system from the body (32).

The Emblok device (*n* = 20 patients) was tested in a prospective, multicenter, non-randomized, first-in-man pilot study intended to evaluate its efficacy and safety during TAVI (32). The device was successfully positioned in all the cases, and no major adverse cardiovascular and cerebrovascular events occurred at the 30-day follow-up. Significant debris was captured in 18 (90%) filters, but 19 (95%) patients had new brain lesions at postprocedural DW-MRI. The median number of new lesions per patient was 10.00 (interquartile range [IQR]: 4.75 to 15.25), the total new lesion volume was 199.9 mm^3^ (IQR: 83.9 to 447.5 mm^3^), and the mean lesion volume per lesion was 42.5 mm^3^ (IQR: 21.5 to 75.6 mm^3^).

### ProtEmbo^®^ cerebral protection system

The ProtEmbo^®^ Cerebral Protection System (Protembis GmbH, Aachen, Germany) is a temporary, intra-aortic embolic deflection filter used as an adjunct device during transcatheter heart interventions and is the only available device that can be positioned through a 6-Fr left radial access (33, 34). ProtEmbo is designed to provide complete cerebral protection and inserted in the beginning of the procedure prior to the TAVI device and removed following the completion of the TAVI procedure. The device consists of (1) a heparin-coated, 60-μm-pore size mesh (currently the smallest pore size of CEPDs), (2) a self-expanding nitinol frame that when expanded ensures sufficient coverage of all cerebral vessels of the aortic arch and includes radiopaque markers for fluoroscopic visualization and precise device placement, and (3) a delivery unit. The device is delivered unexpanded and deployed by unsheathing the self-expanding filter. A handle provides a simple user interface for preparation, delivery, deployment, and removal of the device. The device is loaded into a commercially available delivery catheter and placed into the aortic arch using a commercially available guiding sheath via the left radial artery (33, 34).

The first-generation ProtEmbo device was shown to be safe and feasible in the first-in-human PROTEMBO SF trial (*n* = 4 patients) in two clinical sites in Europe (33). The PROTEMBO C trial (*n* = 41 patients) was a prospective, non-randomized, multicenter (eight sites in Europe) study designed to evaluate the safety and performance of the second-generation ProtEmbo Cerebral Protection System in patients undergoing TAVI (34). The primary safety endpoint was the rate of major adverse cardiac and cerebrovascular events (MACCE) at 30 days, as per the Valve Academic Research Consortium 2 definition, and the primary performance endpoint was the composite rate of technical success compared with performance goals (33). Secondary analyses included the brain DW-MRI new lesion volume and rate of death, or all strokes compared with historical data. Both primary endpoints were met early in this study. MACCE at 30 days were 8.1% (3/37) (upper limit of the 95% confidence interval [CI]: 21.3% versus performance goals 25%, *p* = 0.009), and technical success was 94.6% (35/37) (lower limit of the 95% CI: 82.3% versus performance goals 75%, *p* = 0.003). The new DW-MRI lesion volume with ProtEmbo was lower than that in historical data, and most patients who completed the MRI follow-up (87%, 27/31) were free of any single lesion larger than 150 mm^3^.

## Antithrombotic therapy in transcatheter aortic valve implantation

The current European (2) and American (1) guidelines for the management of valvular heart disease have modified their recommendation favoring the use of single antiplatelet therapy (SAPT) over dual antiplatelet therapy (DAPT) in TAVI patients without an underlying indication for oral anticoagulation (OAC), and in OAC alone over the association of OAC with antiplatelet therapy for TAVI patients requiring lifelong OAC. SAPT on top of vitamin K antagonists (VKAs) may be beneficial only in specific subsets (i.e., TAVI patients with recent acute coronary syndrome or recent coronary stenting). VKA or a direct-acting oral anticoagulant (DOAC) may be considered if OAC is indicated in the absence of contraindications. VKA is indicated in cases of clinical valve thrombosis, accompanied with symptoms or high transvalvular gradient, whereas its role in asymptomatic patients or with those with a normal transvalvular gradient (subclinical leaflet thrombosis) is currently not yet defined. A consensus document of the European Society of Cardiology Working Group on Thrombosis and the European Association of Percutaneous Cardiovascular Interventions (35) supports these recommendations.

### Periprocedural antithrombotic management

The decision to start the antithrombotic therapy before TAVI is not standardized and is primarily left to the physicians’ discretion. However, preprocedural DAPT with aspirin and clopidogrel has been linked to a two-fold increased risk of in-hospital bleeding and transfusions compared with SAPT or no antiplatelet medication, with no clear benefit in terms of ischemic protection (36, 37). Low-dose aspirin is the recommended pre-TAVI treatment in patients without OAC indication (35). Although most patients with TAVI have high residual platelet reactivity to clopidogrel (9, 38), no additional benefits on thromboembolic event reduction have been demonstrated with clopidogrel maintenance or with loading dose prior to TAVI (39).

Among patients on OAC, both VKAs and DOACs are usually stopped before the procedure. A bridging strategy with low-molecular weight heparin is optional and, based on local practice, is restarted for OAC after an uncomplicated intervention (36, 37). Recent evidence suggests that TAVI in patients with OAC may be as safe as and equivalent to an OAC interruption strategy (40–42).

During the intervention, unfractionated heparin is the most used strategy and may be reversed with protamine sulfate at procedure completion according to local practice (43). Although the use of protamine to reverse unfractionated heparin (UFH) after the procedure is not widespread in all TAVI centers and small studies have found no benefit in its use (44), some other evidence points in favor of this strategy after the procedure. In a prospective observational study of 873 patients undergoing TAVI (43), authors found lower rates of the primary composite outcome (a composite of 30-day all-cause mortality and major and life-threatening bleeding) in the group with UFH reversal using protamine (3.2%) than in the control group without heparin reversal (8.7%; *p* = 0.003). This finding was driven by a reduction in major and life-threatening bleeding complications (1.0 vs. 4.1%; *p* = 0.008; and 0.1 vs. 2.6%; *p* < 0.001, respectively). Also, in the control group, the hemoglobin level at 24 h was lower, need for transfusion was higher, and hospital stay was longer, suggesting the benefits for the prevention of clinically relevant complications by protamine administration. Another relevant observation was that thromboembolic complications were equal between the groups. These data are reassuring regarding one of the main concerns of protamine use, which are thrombotic complications, primarily at the transcatheter valve level or in patients with a recent coronary stent. The use of protamine was independently associated with the reduction of the primary composite outcome in the multivariate analysis. Hence, the EAPCI states that protamine sulfate may be used before vascular access closure to reverse anticoagulation with UFH to prevent vascular access site complications and bleedings (35).

The role of procedural bivalirudin is limited to patients who are unable to receive heparin (i.e., allergy and heparin-induced thrombocytopenia) (45). Ongoing studies [Periprocedural Continuation Versus Interruption of Oral Anticoagulant Drugs During Transcatheter Aortic Valve Implantation trial (POPular PAUSE TAVI), NCT04437303] will provide more evidence on this topic.

### Antithrombotic management after transcatheter aortic valve implantation

From the latest ACC/AHA guidelines (1), a SAPT of aspirin (75–100 mg daily) is recommended after TAVI in the absence of other indications for oral anticoagulants (class of recommendation: 2a, level of evidence: B-R), while DAPT (aspirin 75–100 mg plus clopidogrel 75 mg daily) for 3 to 6 months has been retroceded to class of recommendation 2b.

**FIGURE 3:**
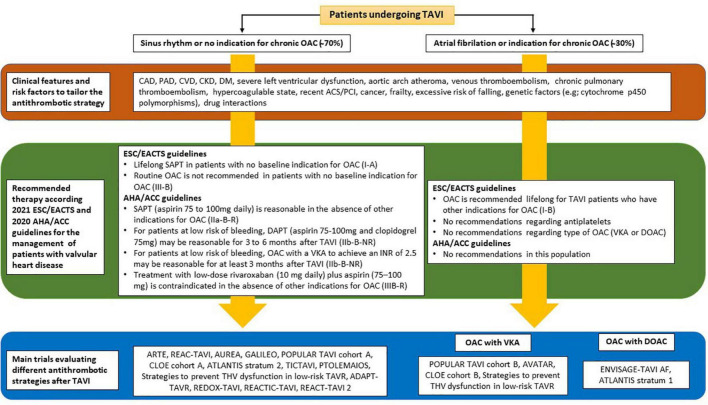
Summary of current recommendations of the European and American guidelines on the antithrombotic regimen after TAVI, including the main studies assessing a variety of antiplatelet and anticoagulant combinations. TAVI, transcatheter aortic valve implantation; OAC, oral anticoagulation; CAD, coronary artery disease; PAD, peripheral artery disease; CVD, cerebrovascular disease; CKD, chronic kidney disease; DM, diabetes mellitus; ACS, acute coronary syndrome; PCI, percutaneous coronary intervention; ESC, European Society of Cardiology; EACTS, European Association for Cardio-Thoracic Surgery; AHA, American Heart Association; ACC, American College of Cardiology; ASA, acetylsalicylic acid; DAPT, dual antiplatelet therapy; SAPT, single antiplatelet therapy; HBR, high-bleeding risk; VKA, vitamin K antagonist; LBR, low bleeding risk; DOAC, direct oral anticoagulant.

In the same line, in the ESC/EACTS guidelines (2), lifelong SAPT is recommended after TAVI in patients with no baseline indication for OAC (class of recommendation: I, level of evidence: A), while the routine use OAC is not recommended after TAVI in patients with no baseline indication for OAC (class of recommendation: III, level of evidence: B) (Figure 3).

Previous observational studies and recent randomized control trials have demonstrated in patients undergoing TAVI with no underlying indication of OAC, the use of DAPT has no advantage over SAPT for the prevention of ischemic events and increases the risk of bleeding. A total of three small-scale RCTs did not find differences between DAPT and SAPT after TAVI on ischemic outcomes (46–48). In the POPular TAVI trial cohort A (49), 665 patients undergoing TAVI without an indication for OAC were randomized to aspirin 100 mg or aspirin 100 mg + clopidogrel 75 mg for 3 months following TAVI (no loading dose prior TAVI). At 1 year, bleeding and the composite endpoint of bleeding or thromboembolic events were significantly less frequent with aspirin monotherapy than with DAPT (15.1 vs. 26.6%, respectively, relative risk [RR] 0.57; 95% CI: 0.42–0.77; *p* = 0.001 for bleeding); and non-procedure-related bleeding (15.1 vs. 24.9%; RR, 0.61 [95% CI, 0.44–0.83]; *p* = 0.005), and this benefit was driven by less major bleeding events, mostly due to periprocedural bleeding. In addition, the rates of ischemia events and valve function measurements were comparable in both groups. However, there are certain scenarios, where in the absence of increased bleeding risk, DAPT should be considered for a limited period (i.e., within 1–12 months), such as recent acute coronary syndrome, complex coronary stenting prior TAVI or during TAVI (chimney stenting), valve-in-valve procedures, large aortic arch atheromas, and previous non-cardioembolic stroke.

Also, the use of OAC (either VKA or DOAC) has not shown evidence to support its use. The GALILEO trial (*n* = 1,644 patients) tested rivaroxaban 10 mg/d (plus aspirin for the first 3 months) versus aspirin 75 to 100 mg/d (plus clopidogrel 75 mg/d for the first 3 months). The authors found a higher risk of thromboembolic complications (hazard ratio [HR], 1.35 [95% CI, 1.01–1.81]; *p* = 0.04), death (HR, 1.69 [95% CI, 1.13–2.53]), and major, disabling, or life-threatening bleeding (HR, 1.50 [95% CI, 0.95–2.37]; *p* = 0.08) with the OAC strategy (50). Notably, in the GALILEO-4D substudy (*n* = 231), patients treated with rivaroxaban plus aspirin showed a less frequency of subclinical leaflet motion anomalies and leaflet thrombosis than patients treated with a DAPT regimen (51). The ADAPT-TAVR (*n* = 229) was an open-label trial that evaluated the use of edoxaban for 6 months or DAPT with ASA plus clopidogrel on leaflet thrombosis assessed by 4DCT in patients without indication of OAC. At 6 months after TAVI, the researchers noted no link between subclinical leaflet thrombosis and an increased risk of cerebral thromboembolism or neurological impairment (52). Also, no statistically significant difference between edoxaban and DAPT in leaflet thrombosis incidents were found, although edoxaban group patients did show a lower trend (9.8 vs. 18.4% for DAPT; absolute difference: −8.5%; 95% CI: −17.8 to 0.8%; *p* = 0.076). The edoxaban group had numerically more new cerebral lesions on DW-MRI than the DAPT group (25.0 vs. 20.2%, respectively; difference, 4.8%; 95% CI: −6.4 to 16.0%; *p* = 0.40). The median total new lesion number (1 for each group; *p* = 0.85) and volume (36.6 mm^3^ for edoxaban and 43.9 mm^3^ for DAPT; *p* = 0.88) were also not different between the two groups. Neurocognitive outcomes measured by the NIHSS, modified Rankin Scale, and Montreal Cognitive Assessment, and any or major bleeding events (11.7% of the edoxaban patients versus 12.7% on DAPT, hazard ratio 0.93; 95% CI: 0.44–1.96) were comparable between the two groups. Similar data on the potential lack of benefit on the prevention of silent cerebral lesions after TAVI with OAC (acenocoumarol) compared with DAPT (aspirin + clopidogrel) has been presented (53).

For patients undergoing TAVI with underlying indication of long-term OAC, definitive evidence supporting DOACs over VKAs after TAVI is currently lacking. Observational data have shown inconsistent results regarding the thromboembolic risk associated with DOACs in the post-TAVI population. A collaborative registry between German and Italian centers (*n* = 962) showed higher all-cause mortality, myocardial infarction, and cerebrovascular events at 1 year with DOACs (rivaroxaban, apixaban, or dabigatran) than with VKA, with a comparable 1-year event rates of bleeding (54), while in a nationwide observational cohort Danish study (*n* = 735), a similar risk of thromboembolism, bleeding, or all-cause mortality post-TAVI among DOACs (dabigatran, rivaroxaban, apixaban, or edoxaban) and VKAs (warfarin or phenprocoumon) was found (55). According to the data from the PARTNER 2 cohort, OAC alone was ineffective in reducing 2-year stroke, while antiplatelet therapy with or without anticoagulant therapy significantly lowered the risk of stroke at 2 years after TAVI. OAC, on the other hand, was linked to a lower risk of combined death and stroke when taken alone (56).

The POPular TAVI cohort B (*n* = 326) evaluated the safety and efficacy of OAC plus clopidogrel or OAC alone post-TAVI (57). The rate of non-procedural bleeding at 1 year was considerably higher in the OAC plus clopidogrel than in the OAC alone group (34 vs. 21.7%, *p* = 0.02), while the composite of cardiovascular death, stroke, or MI was comparable between the two treatment strategies non-inferior (17.3 and 13.4%, respectively; 95% CI for non-inferiority, −11.9 to 4.0).

The ENVISAGE-TAVI AF (*n* = 1,426) trial compared edoxaban with VKAs in patients with an indication for anticoagulation (58). Regarding NACE (death from any cause, myocardial infarction, ischemic stroke, systemic thromboembolism, valve thrombosis, or major bleeding), edoxaban was non-inferior to VKA (17.3 vs. 16.5 per 100 person-years; HR, 1.05; 95% CI, 0.85 to 1.31; *p* = 0.01 for non-inferiority), but edoxaban was associated with a higher incidence of major bleeding (mostly gastrointestinal bleeds) than VKA, mainly among patients who received specified concomitant antiplatelet therapy (9.7 vs. 7.0 per 100 person-years; HR, 1.40; 95% CI, 1.03 to 1.91; *p* = 0.93 for non-inferiority). No valve thrombosis events were reported in the trial.

The recently published ATLANTIS trial (*n* = 1,500) tested apixaban 5 mg (2.5 mg if impaired renal function or concomitant antiplatelet therapy) (*n* = 749) two times daily, or standard of care (*n* = 751) (59). In stratum 1, patients in the standard-of-care group received a VKA, while in stratum 2, patients received antiplatelet therapy with aspirin and clopidogrel, if there was an indication for anticoagulation or not, respectively. The primary endpoint was the composite of death, myocardial infarction, stroke or transient ischemic attack, systemic embolism, intracardiac or bioprosthesis thrombosis, deep vein thrombosis or pulmonary embolism, and life-threatening, disabling, or major bleeding over the 1-year follow-up. The primary safety endpoint was major, disabling, or life-threatening bleeding. Apixaban was not superior to standard of care globally (18.4 vs. 20.1%; HR 0.92; 95% CI 0.73–1.16; P interaction = 0.57) and in each stratum arms (indication or not for OAC). Similar to observed in the GALILEO trials (50, 51), subclinical valve thrombosis was reduced with apixaban compared with the aspirin and clopidogrel regimen (HR 0.19; 95% CI 0.08–0.46), while a signal of higher non-cardiovascular mortality was observed with apixaban.

Ongoing trials (AVATAR, NCT02735902; Strategies to Prevent Transcatheter Heart Valve Dysfunction in Low Risk Transcatheter Aortic Valve Replacement, NCT03557242; REACTIC-TAVI, NCT04331145; REAC-TAVI 2, NCT05283356; and REDOX-TAVI, NCT04171726) will provide more information regarding the antithrombotic management on this complex field.

The conventional TAVI population carries a large burden of comorbidities that make them more susceptible to long-term cerebrovascular events. The incidence of diabetes mellitus, coronary artery disease, history of atrial fibrillation, previous stroke, or peripheral vascular disease raises up to 60–70% in TAVI recipients, including high- to intermediate-risk (60–64) to low-risk patients (65, 66).

In these patients, achieving an optimal long-term antithrombotic strategy that provides protection from future ischemic events (stroke, myocardial infarction, or valve thrombosis) without significantly increasing the cumulative risk of bleeding over time is crucial. This long-term treatment is very relevant primarily in the low-risk population and in younger patients with a long life expectancy in whom extending the durability of the aortic bioprosthesis as much as possible is essential to avoid repeat interventions, as well as in certain scenarios, such as bicuspid valve, valve-in-valve, or valve-in-TAV procedures. Also, it should be in line with the optimal medical management of their comorbidities. Some studies are in this direction (NCT05283356, NCT03042104, NCT02825134, NCT03972644, NCT04204915, and NCT03094143), exploring the lifetime management of the TAVI population and will provide data in future.

## Conclusion

In current TAVI practice, the rate of overt stroke during or early after TAVI is relatively low (2–4%) (5) but remain stable over the years (4). However, it may represent only the tip of the iceberg of cerebral cardioembolic events, being microembolization and cerebral “silent” injury more frequent phenomena, but still poorly understood with a potential substantial impact on mid- and long-term cognitive function. Although there is still not enough clinical evidence to conclusively establish a direct relationship between the use of CEPDs and stroke prevention, the available studies point to significant protection from periprocedural cerebrovascular events. Important studies are under way to clarify this point (NCT04149535 and ISRCTN16665769). Therefore, in light of TAVI expansion to lower risk patients and the younger population, measures to abate neurological risks during and after TAVI are warranted.

## Author contributions

VJ, RE, and AI developed the concept, design, and drafted the manuscript. JB, PJ, GB, BC, and CV contributed substantially to the critical revision of the manuscript and add important intellectual content. All authors contributed to the article and approved the submitted version.
